# Unconventional Source of Neurotoxic Protein Aggregation from Organelle Off-Target Bax∆2 in Alzheimer’s Disease

**DOI:** 10.3390/biom13060970

**Published:** 2023-06-10

**Authors:** Qi Yao, Anne Caroline Mascarenhas dos Santos, Huaiyuan Zhang, Adriana Mañas, Ammarah Hussaini, Ujin Kim, Congtai Xu, Sana Basheer, Shinya Tasaki, Jialing Xiang

**Affiliations:** 1 Department of Biology, Illinois Institute of Technology, Chicago, IL 60616, USAcxu40@hawk.iit.edu (C.X.);; 2 Department of Laboratory Medicine, Lund University, 22381 Lund, Sweden; 3 Rush Alzheimer’s Disease Center, Rush University Medical Center, Chicago, IL 60612, USA

**Keywords:** Bax, Bax∆2, Alzheimer’s disease, aggregates, stress granules, cell death

## Abstract

Protein aggregates are a hallmark of Alzheimer’s disease (AD). Extensive studies have focused on β-amyloid plaques and Tau tangles. Here, we illustrate a novel source of protein aggregates in AD neurons from organelle off-target proteins. Bax is a mitochondrial pore-forming pro-death protein. What happens to Bax if it fails to target mitochondria? We previously showed that a mitochondrial target-deficient alternatively spliced variant, Bax∆2, formed large cytosolic protein aggregates and triggered caspase 8-mediated cell death. Bax∆2 protein levels were low in most normal organs and the proteins were quickly degraded in cancer. Here, we found that 85% of AD patients had Bax∆2 required alternative splicing. Increased Bax∆2 proteins were mostly accumulated in neurons of AD-susceptible brain regions. Intracellularly, Bax∆2 aggregates distributed independently of Tau tangles. Interestingly, Bax∆2 aggregates triggered the formation of stress granules (SGs), a large protein-RNA complex involved in AD pathogenesis. Although the functional domains required for aggregation and cell death are the same as in cancer cells, Bax∆2 relied on SGs, not caspase 8, for neuronal cell death. These results imply that the aggregation of organelle off-target proteins, such as Bax∆2, broadens the scope of traditional AD pathogenic proteins that contribute to the neuronal stress responses and AD pathogenesis.

## 1. Introduction

Bax is a pro-death member of the Bcl-2 family, and plays crucial roles in a wide range of physiological and pathological processes [1,2,3]. Mitochondria are the direct target of Bax-mediated cell death. Cytosolic monomeric Bax (specifically, Baxα relies on oligomerization to form a “pore” on the mitochondrial outer membrane for apoptotic cell death [4]. Although Bax does not contain a well-defined mitochondrial leading sequence, the functional domains responsible for targeting mitochondria and cell death are clear. The BAX gene encodes six exons and nine alpha helices. The N-terminal helix α1, encoded by exon 2, is responsible for Bax activation and translocation to mitochondria [2,5]. The C-terminal helix α9, encoded by exon 6, anchors Bax onto the mitochondrial outer membrane [6,7]. The core region of Bax is critical for high-order oligomerization and formation of the pore, which allows release of cytochrome C from the mitochondria and triggers the apoptotic cascade [8,9,10].

In previous studies, we discovered a unique alternative spliced Bax variant, Bax∆2, which is incapable of targeting mitochondria due to lack of exon 2 [11,12]. Bax∆2 contains 10 frameshifted amino acids at the beginning of the exon 3, which allowed us to generate an antibody specific to Bax∆2 without cross-reacting with Baxα [11,12,13]. Other than these elements, Bax∆2 retains the same sequence as Baxα, including the core regions for homodimerization and heterodimerization and pro-death Bcl-2 homology (BH) domains [11,12]. Baxα protein is ubiquitously and readily detected in most normal human organ systems [14,15,16], except for the brain [15,16,17]. In contrast, the distribution of Bax∆2 proteins in normal human tissues was found to be scattered and at lower levels [13]. Bax∆2 protein is unstable in cancer cells due to elevated proteasomal degradation [18]; therefore, it was rarely detected in highly malignant tumors [13].

Can Bax∆2 still cause cell death if it cannot target mitochondria? We have uncovered the mechanism of Bax∆2-mediated cell death using both wet and dry lab approaches [19,20,21]. Although Bax∆2 did not target mitochondria due to the lack of exon 2 encoding helix α1, it contains an intact core region capable of oligomerization [12]. As a result, Bax∆2 proteins formed large protein aggregates in the cytosol which served as a platform to recruit caspase 8 using the C-terminal helix α9 (the mitochondrial anchoring domain for Baxα) [19,21]. Binding of caspase 8 to Bax∆2 aggregates triggered caspase-mediated but mitochondria-independent cell death. The formation of Bax∆2 aggregates was required for both interaction with caspase 8 and cell death since disruption of the Bax∆2 core region diminished the protein aggregation. Without the aggregate, there was no caspase 8 recruitment and no cell death [19]. Notably, the Bax∆2 C-terminal helix α9 acted as a “bridge” between Bax∆2 aggregates and caspase 8 interaction. Without the helix α9, Bax∆2 protein still formed aggregates, but such aggregates could not interact with caspase 8 to trigger cell death [19,21]. This suggests that protein aggregation alone is essential but not sufficient for the execution of cell death.

The pathological role of the Bax∆2 protein aggregates in disease remains to be explored. After studying Bax∆2 in cancer cells, we wondered whether these Bax∆2 protein aggregates could contribute to neurodegenerative diseases, particularly Alzheimer’s disease (AD), a progressive neurodegenerative disorder hallmarked by misfolded protein aggregates [22,23]. Most of the studies in this field have extensively focused on the aggregations of intracellular Tau protein and extracellular amyloid beta protein [24,25,26,27]. It has been documented that Bax proteins were detected in some AD brains [28,29], and increased in tau tangle-positive cells [30,31], suggesting that Bax may play a role in AD. However, it is still largely questioned whether neuronal death is apoptosis, especially Bax-mitochondria-dependent apoptosis. We hypothesized that Bax∆2 aggregates may provide an alternative pathway independent of Bax-mitochondria in the contribution to AD neuronal death. Therefore, studying the cellular behavior of Bax∆2 aggregates in neurons could provide us with new insight into an alternative source of protein aggregates that contribute to AD pathogenesis.

## 2. Materials and Methods

### 2.1. Materials

The postmortem human brain formalin-fixed paraffin-embedded tissue sections for immunostaining were obtained from BioChain (Newark, CA, USA), Biomax (Derwood, MD, USA), and Rush Alzheimer’s Disease Center (RADC, Chicago, IL, USA). The frozen postmortem human brain tissues for immunoblotting were from BioChain. This study was granted with exemption by the Institutional Review Board (IRB) at the Illinois Institute of Technology (Legacy-IRB-2019-017). Antibodies against total-Tau (T-Tau; D1M9X), phosphorylated Tau (P-Tau; T181), P-Tau (T205), TIA-1, and Bax (D2E11) were purchased from Cell Signaling Technology (Danvers, MA, USA); Bax (N20) was purchased from Santa Cruz Biotechnology (Dallas, TX, USA). The Bax∆2 monoclonal antibody (2D4), generated against amino acids (GFHGSSRANG) unique to Bax∆2, is well characterized, and was confirmed not to cross-react with Baxα in either immunostaining or immunoblotting [13,32].

### 2.2. RNA-Seq Mapping and Analysis

AD brain RNA-seq datasets (ID: syn24175555 and syn8612097) [33] together with clinical data and biospecimen metadata files from the ROSMAP study [34,35] (ROSMAP_clinical.csv, ROSMAP_CognitiveResilience_biospecimen_metadata.csv and ROSMAP_biospecimen_metadata.csv, respectively) were retrieved from the AD Knowledge Portal [36]. RNA-seq datasets were mapped against the Baxα and Bax∆2 reference genes (GenBank accession numbers AAA03619 and AFU81108, respectively) with minimap2 v2.21-r1071 [37] using get_SNPs.pl v2.0 from the SSRG pipeline (https://github.com/PombertLab/SSRG, accessed on 20 March 2023) and the ‘--rmo’, ‘--bam’ and ‘--idx csi’ command line arguments. Percentages of Bax transcripts featuring or missing the exon 2 required for proper localization of mitochondria were calculated from the RNA-seq data that aligned against the Baxα and Bax∆2 sequences with get_BAX.pl v0.1a (the custom script is in the code availability section). Briefly, RNA-seq reads with primary alignments mapping to the corresponding exon junctions in the reference Baxα/Bax∆2 genes (overlapping bases 30 to 40 in Baxα and Bax∆2) were extracted from the BAM alignments, and these subsets were converted to SAM format with SAMtools’s view function v1.13 using the samflag ‘-F 256’ [38]. Each RNA-seq dataset was linked and matched to the corresponding clinical and biospecimen, and the relevant information was summarized with the normalized percentages of Baxα and Bax∆2 in a de-identified tab-delimited file through get_BAX.pl. Data in the SAM and summarized tab-delimited files were de-identified by replacing samples IDs with unlinked numbers and nucleotide sequences with strings of N of equal lengths wherever applicable. Exon junctions were illustrated with BamSnap v0.2.19 [39] as implemented in get_BAX.pl. BAM and SAM files containing ROSMAP metadata identifiers were deleted immediately following computation.

### 2.3. Cell Culture and Transfection

The murine hippocampal neuronal cell line HT22 was obtained from Kerafast (Boston, MA, USA). The Bax-negative HCT116 subline cell was generated as described previously [11,13]. All cells were cultured in Dulbecco’s modified Eagle’s medium (DMEM) with 10% fetal bovine serum (FBS). For transfection, cells were split into 6-well plates and allowed to grow to approximately 60% confluence and then transfected with different constructs (GFP, green fluorescent protein), Bax∆2, or its derived constructs (Bax∆2 [∆BH3] and Bax∆2[∆α9]) [19] using Lipofectamine 3000 reagent from Invitrogen (Waltham, MA, USA). Cells were then incubated for the appropriate time corresponding to each experiment before downstream analysis.

### 2.4. Cell Death Assay

Bax-negative HCT116 subline cells or HT22 cells were transfected with Bax∆2 and its derived constructs (Bax∆2 [∆BH3], Bax∆2[∆E6]) and incubated for 48 h in the absence or presence of 50 μM caspase 8 inhibitor z-IETD-fmk from R&D Systems (Minneapolis, MN, USA). Floating and attached cells were harvested separately and concentrated by a cytospin onto glass slides. Cells were then fixed with 4% paraformaldehyde and permeabilized with 0.2% Triton X-100 in phosphate-buffered saline (PBS). Cells were blocked and incubated with primary antibody against Bax∆2 (2D4, 1:100 in blocking buffer containing 0.2% Triton X-100, 5% bovine serum albumin (BSA) in PBS) overnight at 4 °C, followed by Alexa Fluor conjugated secondary antibodies (Invitrogen, 1:200 dilution in PBS) for 1 h at room temperature. Nuclei were stained with DAPI. Fluorescent images were obtained using a Keyence BZ-X710 All-in-One fluorescence microscope and quantitated by a BZ-X analyzer (Itasca, IL, USA). The number of floating cells expressing Bax∆2 was divided by the total number of Bax∆2-positive cells for the percentage of cell death percentage. The experiments were performed in triplicate.

### 2.5. Immunoblotting

Cell pellets were lysed in NP-40 buffer (145 mM NaCl, 5 mM MgCl_2_, 1 mM EGTA, 0.25% NP-40, 20 mM HEPES, pH 7.4) with a cocktail of protease inhibitors at 4 °C for 30 min. Equal amounts of protein for each sample were loaded into a 12% SDS-PAGE gel and transferred onto a 0.2 μm PVDF membrane. Membranes were blotted with 5% BSA in PBST (0.2% Tween 20 in PBS), followed by incubation with the appropriate primary antibody against Baxα (N20 or D2E11, 1:1000), Bax∆2 (2D4, 1:200), or actin (Millipore, 1:3000 dilution) overnight at 4 °C. The membrane was washed with PBST five times, eight minutes each time. After incubation with HRP-conjugated secondary antibody (Jackson, West Grove, PA, USA) for 1 h followed by five washes, six minutes each time, the protein bands were visualized on a ChemiDOC imaging system (BIO-RAD, Hercules, CA, USA) by a Pierce ECL Western Blotting Substrate kit from ThermoScientific (Waltham, MA, USA), and the results were analyzed by using ImageJ v1.52d.

### 2.6. Immunofluorescence Staining

HT22 cells were transfected with either GFP or different Bax∆2 derived constructs (Bax∆2, Bax∆2[∆E6], Bax∆2[∆BH3]) [19] on glass cover slides in 6-well plates for 24 h. The slides were fixed with 4% paraformaldehyde and permeabilized with 0.2% Triton X-100 in PBS. Cells were incubated with primary antibodies (antibody against Bax∆2 (2D4, 1:100), and a stress granules marker (TIA-1, 1:50) overnight at 4 °C, followed by Alexa Fluor secondary antibodies (Invitrogen, 1:200 dilution) for 1 h at room temperature. Nuclei were stained with DAPI. Fluorescent images were obtained using a Keyence BZ-X710 All-in-One fluorescence microscope and analyzed using ImageJ v1.52d.

### 2.7. Immunohistochemical Staining

Tissue slides were dewaxed using xylene and rehydrated using graded ethanol solutions (100%, 95%, and 75%). Endogenous peroxidase was removed using 3% hydrogen peroxide. Slides were incubated for 10 min in sodium citrate buffer (0.01 M, pH 6.0) at 95 °C for epitope retrieval, followed by blocking (5% BSA, 0.1% Tween 20 in 1× PBS) for 2 h. The slides were then incubated in CoverWell humidity chambers with antibody Bax∆2 (1:200), anti-T-Tau (1:100), P-Tau T181/T205 (1:100) or TIA-1 (1:50) at 4 °C overnight, followed by biotin-conjugated secondary antibody (1:200) at room temperature (RT) for 2 h. For antibody neutralization, the Bax∆2 antibody was pre-incubated with the Bax∆2 antigen peptide (QPRGGGFHPGSSRANGGEAP, 1:100 ratio) or equivalent control Baxα antigen peptide (ALLLQGFIQDRAGRMGGEAP) overnight at 4 °C before applying to tissue slides. A Vectrastain ABC kit (Vector Laboratories, Newark, CA, USA) and ImmPACT DAB Peroxidase Substrate kit (Vector Laboratories) were used for visualization, and Hematoxylin QS (Vector Laboratories) was used for nuclear staining. The slides were dehydrated and fixed by using the xylene-based mounting media Poly-Mount (Polysciences Inc., Warrington, PA, USA). The fluorescence staining procedure was the same as described above in this section until the primary antibody incubation step. Slides were then incubated with Alexa Fluor 488, 597, or 647 secondary antibodies (Invitrogen, 1:200 dilution) at room temperature for 2 h. Slides were sealed with ProLong Gold antifade reagent (Invitrogen).

### 2.8. Image Acquisition, Process, and Quantitation

Immunofluorescence-stained slides were imaged using a CSU-W1 spinning disc confocal microscope with two Hamamatsu ORCA-Flash4.0 cameras at the Center for Advanced Microscopy facility at Northwestern University (Chicago, IL, USA) and visualized using ImageJ. For immunohistochemical staining, the slides were scanned using a CRi Pannoramic Scan Whole Slide Scanner with a 40× NA 0.95 LWD Zeiss objective, on high resolution (down to 0.12 μm/pixel) at the Integrated Light Microscopy Core Facility at the University of Chicago (Chicago, IL, USA), and visualized using Pannoramic Viewer v1.15.4. Stained slides were evaluated using software image analysis. Individual captured image selections were processed using CellProfiler v2.2.0 [40], which can quantitate the total number of positive cells in reference to the number of nuclei. For neuronal nuclei, the parameters were: input (hematoxylin), diameter of objects (12–42), smoothing filter (3); for positive stained neurons, the parameters were input (threshDAB), diameter of objects (34–44), threshold (0.135), and smoothing filter (10). For non-neuronal cell nuclei, such as glial cells and granule cells, the parameters were: input (hematoxylin), diameter of objects (5–11), and smoothing filter (5); for non-neuronal-positive cells, the parameters were: input (threshDAB), diameter of objects (5–11), threshold (0.45), and smoothing filter (10). At least 50% of the entire area of the stained tissue was selected and analyzed. The edges of each tissue section were generally not subjected to analysis to avoid false positives from DAB precipitation.

### 2.9. Statistics

All statistical analyses were performed using GraphPad Prism v9. All values were expressed as the mean ± standard deviation (SD). The Student *t*-test or Mann–Whitney test were used to compare two groups unless otherwise stated. Comparisons of more than two groups were analyzed with either one-way or two-way ANOVA followed by Tukey’s multiple comparisons test. *P* values under 0.05 were considered statistically significant (*, *p* < 0.05. **, *p* < 0.01. ***, *p* < 0.001. ****, *p* < 0.0001).

## 3. Results

### 3.1. Bax Exon 2 Alternative Splicing Is Significantly Higher in AD Brains

**Figure 1:**
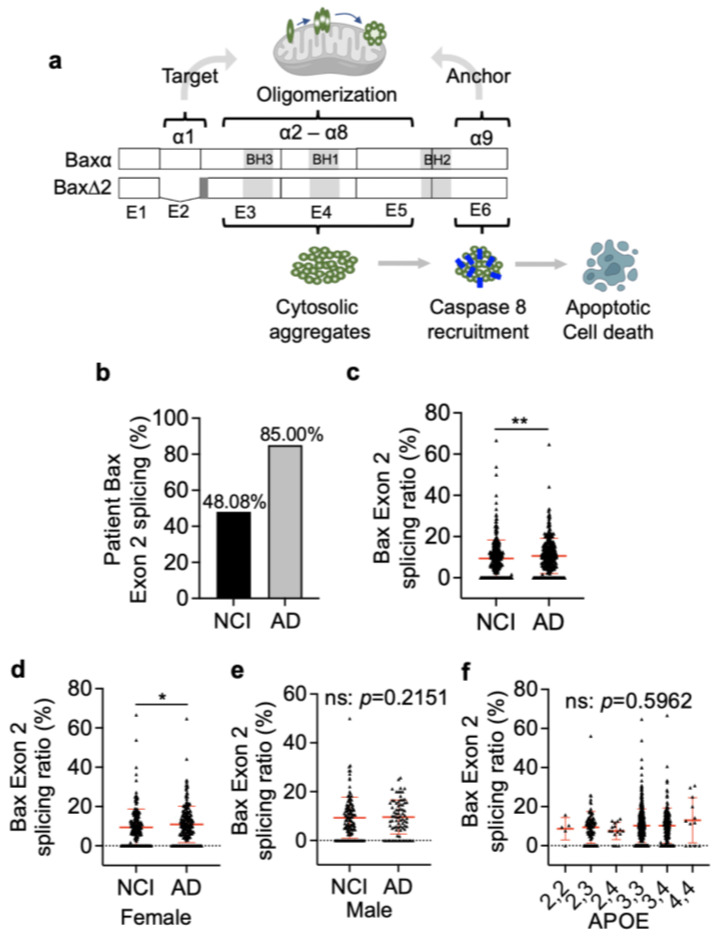
Analysis of Bax exon 2 alternative splicing in Alzheimer’s brain tissue using RNA-seq. (**a**) An overview of the differences between Baxα and Bax∆2. Bax exons are labeled as E1 to E6. The corresponding helices are labeled on the top. The dark grey in the beginning of exon 3 is the frameshifted region for Bax∆2. (**b**) The percentage of patients who had Bax exon 2 splicing within the RNA-seq data examined. NCI, no cognitive impairment (n = 327); AD, Alzheimer’s disease (n = 324). (**c**) Quantitation of the ratio of Bax transcripts with exon 2 splicing over the total Bax transcripts in individual NCI (n = 327) and AD patients (n = 324), analyzed using the Mann–Whitney test. **, *p* < 0.01. The ratio of exon 2 splicing in relation with AD patients’ genders (**d**) female (NCI, n = 209; AD, n = 210), (**e**) male (NCI, n = 117; AD, n = 78), and (**f**) APOE genotypes (22, n = 5; 23, n = 96; 24, n = 16; 33, n = 515; 34, n = 171; 44, n = 12) were analyzed via Mann–Whitney test and one-way ANOVA, respectively. ns, not statistically significant; *, *p* < 0.05, **, *p* < 0.01.

**Figure 2:**
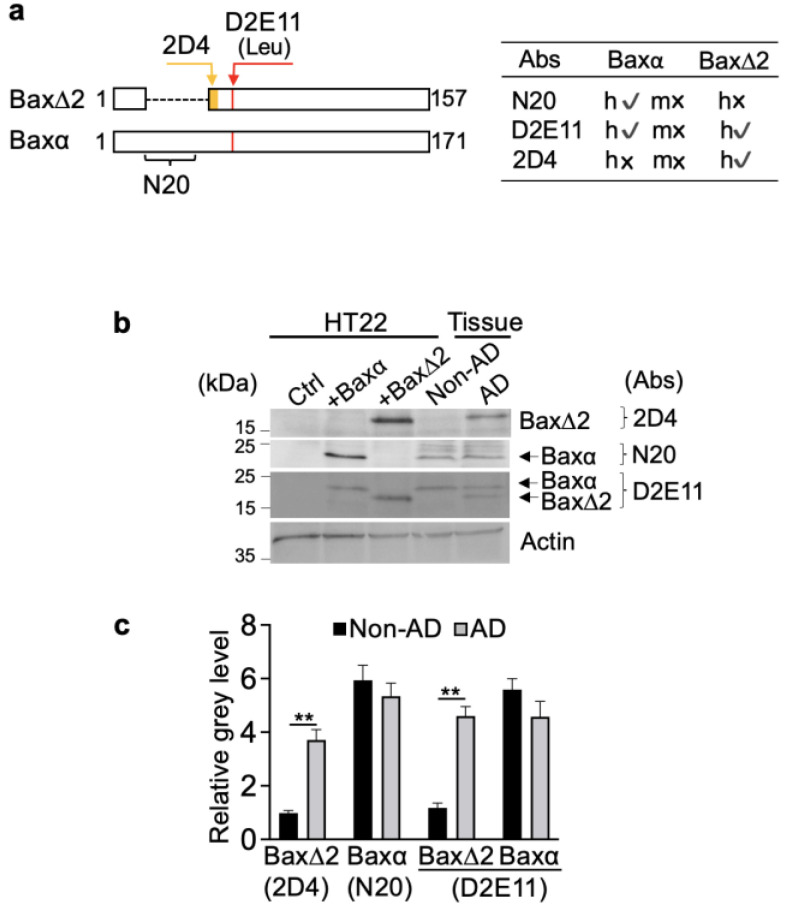
Detection of Bax∆2 protein in AD brain tissue. (**a**) The antigen epitopes of different Bax antibodies and their antibody cross-reactivities. h, human; m, mouse; √ indicates the antibody reacts with the species; x indicates the antibody does not react with the species. (**b**) Immunoblots and (**c**) quantitation of ectopically expressed human Bax∆2 and Baxα in HT22 mouse hippocampus neuronal cells, and endogenous Bax∆2 and Baxα in non-AD (and 82-year-old male) and AD (85-year-old female) brain frontal lobe tissues using anti-Bax∆2 antibody (2D4), anti-Baxα (N20) antibody, and anti-Bax∆2/Baxα antibody (D2E11); actin was used as protein-loading control. Statistical significance was measured using the Mann–Whitney test, **, *p* < 0.01.

Unlike Baxα, Bax∆2 cannot target mitochondria, and its aggregates rely on caspase 8 to trigger cell death (Figure 1a). As the removal of Bax exon 2 by alternative splicing is required to produce Bax∆2, profiling a cohort of AD patients at the transcript levels could provide us with more information. We used RNA-seq databases from the Religious Orders Study and Memory and Aging Project (ROSMAP) containing frontal lobe transcriptomes from 324 AD and 327 no cognitive impairment (NCI) controls (Figure S1). We specifically focused on the transcript splicing junction between exon 1 and 2 of Baxα. The results showed that about 85% of the AD frontal lobes contained Bax exon 2 alternatively spliced transcripts, about 40% more than that in the NCI control group (Figure 2a). The ratio of Bax transcripts without exon 2 over the total Bax transcripts was statistically significantly higher in the AD group than the control (*p* = 0.0096) (Figure 2b), mostly contributed by female AD patients (Figure 2c), not male patients (Figure 2d). The Bax exon 2 splicing had no statistical correlation with the AD patient’s APOE genotypes (apolipoprotein E, especially APOE4, is a well-documented genetic risk factor for Alzheimer’s disease) [41] (Figure 2e). The increased Bax exon 2 alternative splicing in AD was consistent with our observation of increased Bax∆2 protein level (Figure 1c). These results suggest that Bax exon 2 splicing is increased in AD patients and possibly contributes to Bax∆2 protein production in AD brain tissue.

### 3.2. Bax∆2 Protein Accumulated in Neuronal Cells from AD-Affected Brain Regions

To examine Bax∆2 protein levels, we obtained one pair of frozen tissues (brain frontal lobe from a postmortem 85-year-old female AD and 82-year-old male non-AD patient). For immunoblot, antibodies against different region of Baxα and Bax∆2 were illustrated in Figure 2a. Ectopic expression of Baxα or Bax∆2 in HT22 mouse hippocampal neuronal cells was used as controls. HT22 cells do not contain endogenous Bax∆2. The antibody against Bax∆2 (2D4) was derived from a unique peptide which does not exist in Baxα (Figure 2a). Of note, Bax∆2 antibody has been extensively characterized and has shown no cross reactivity with Baxα. We further validated the antibody in this study (Figure S2). The epitope for Baxα N20 antibody is mainly located in the exon 2 and does not exist in Bax∆2. The N20 antibody only recognizes human and not murine Bax. The epitope for Bax D2E11 antibody is shared by both Baxα and Bax∆2. The immunoblot results showed that Bax∆2 protein was barely detected in the non-AD brain tissue, as expected, but the level significantly increased in the AD brain tissue (Figure 2b). In contrast, Baxα protein remained at similar levels in both non-AD and AD samples (Figure 2b). These results suggest that Bax∆2, but not Baxα protein was selectively increased in AD brain tissue examined here, but further investigation is needed to confirm this notion.

**Figure 3:**
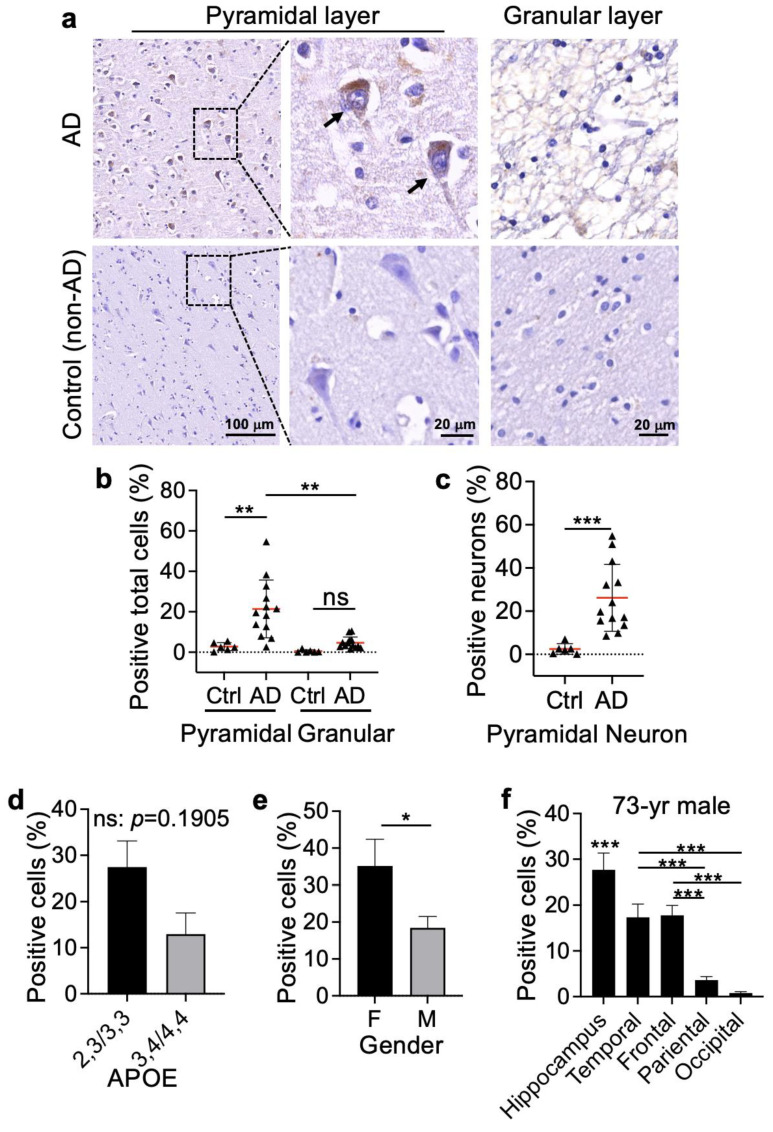
Detection and distribution of Bax∆2-positive cells in Alzheimer’s brain tissue. (**a**) Human brain frontal lobe tissue was immunohistochemically (IHC) stained with anti-Bax∆2 antibody. Images from two individuals (the non-AD subject, 54-year-old male; the AD subject, 73-year-old male) are shown. The boxed regions are enlarged on the right for the pyramidal layer. Scale bar, 100 µm for the first column of images. Scale bar, 20 µm for the second column of images. (**b**,**c**) The total positive cells in the pyramidal and granular layers (**b**) and the positive neurons in the pyramidal layer (**c**) were quantified in the control (Ctrl, n = 6) and AD patients (n = 13). (**d**,**e**) Association of Bax∆2 protein levels with APOE genotypes (**d**) (23, n = 1; 33, n = 4; 34, n = 3; 44, n = 1) and gender (F, n = 6; M, n = 7) (**e**) in AD patients. (**f**) Quantitation of Bax∆2 IHC staining of the different brain regions as indicated from an AD patient (73-year-old male). Two-way ANOVA was used for (**b**); the Mann–Whitney test was used for (**c**–**e**); and one-way ANOVA for (**f**), the hippocampus was compared to all different lobes from a 73-year-old male AD patient. *, *p* < 0.05; **, *p* < 0.01; ***, *p* < 0.001; ns, not statistically significant.

To further confirm the observation above, we immunobiologically stained frontal lobe tissue from a cohort of AD patients (n = 13) and non-AD controls (n = 6) using a Bax∆2-specific antibody (the patient information is listed in Figure S3). The results showed that Bax∆2 positively stained cells were easily visible in AD samples in contrast to the non-AD controls (Figure 3a). Most positive cells were located in the pyramidal layer rather than the granular layer in the frontal lobe (Figure 3b). Morphologically, most of the Bax∆2-positive staining was concentrated in the neuronal body. The number of Bax∆2-positive neurons varied widely among individual AD samples (9–55%) with an average of 27% compared to a narrower range in the control group (0–18%, average 3%) (Figure 3c). Similar to the results from the RNA-seq analysis, increased Bax∆2 protein levels did not statistically correlate with APOE genotypes in the AD group (Figure 3d) but were significantly higher in female AD patients (Figure 3e). In addition, we performed correlation analyses between Bax∆2 and different ages in both male and female AD groups (Figure S4), and we did not observe notable correlation, taking into the consideration the small sample size used here. Altogether, these results suggest that Bax∆2 was upregulated in the neurons of AD-affected brain regions.

We also obtained different brain region tissues from one AD individual (73-year-old male) and further investigated the distributions of Bax∆2-positive cells in different AD-susceptible brain regions. The results showed that Bax∆2-positive detection had the highest levels in the hippocampus (28%), followed by the frontal and temporal lobes (17% for both) but nearly non-detectable in the parietal and occipital lobes (1–3%) (Figure 3f and Figure S5). Control samples of the same regions were mainly negative or weakly positive (Figure S6). Although the data were from a single AD patient, these results imply that Bax∆2 distribution may be regionally selective and, at least in this case, is consistent with AD pathology.

### 3.3. Bax∆2 Aggregate Distribution Is Independent of Tau Tangles in AD Neurons

**Figure 4:**
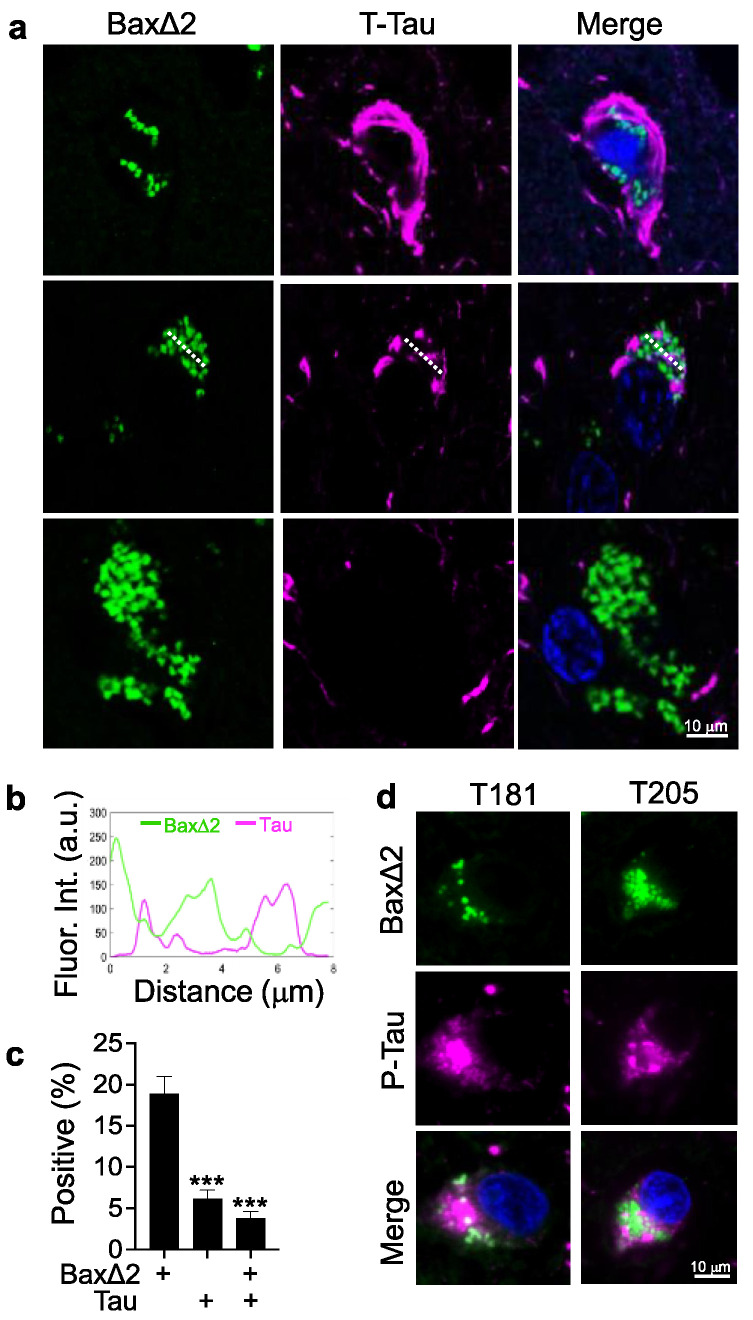
Intracellular distribution of Bax∆2 aggregates in relation to Tau tangles in AD neurons. (**a**) Co-immunofluorescence staining of AD frontal lobe tissues with anti-Bax∆2 (green) and anti-total Tau (purple) antibodies. Nuclei were stained with DAPI. Scale bar, 10 μm. (**b**) Line scan of fluorescence intensity from the image indicated in (**a**), second row. (**c**) Quantitation of single- and double-positive neurons for Bax∆2 and Tau in the brain tissue of two AD patients (73-year-old male and 85-year-old female) using the Mann–Whitney test. ***, *p* < 0.001. (**d**) Co-immunofluorescence staining of AD frontal lobe tissue with anti-Bax∆2 (green) and anti-P-Tau (T181 and T205; purple) antibodies. Nuclei were stained with DAPI. Scale bar, 10 μm.

To investigate the intracellular distribution of Bax∆2 aggregates and their relationship with the AD hallmark Tau tangles, we examined cellular localization of both Bax∆2 and T-Tau proteins by co-immunostaining in the AD frontal lobe tissues of a 73-year-old male patient. The results showed that strong positive staining of both Tau tangles and Bax∆2 aggregates were easily detected in the AD pyramidal neurons in the pyramidal layer (Figure 4a). The protein distribution pattern of Bax∆2 aggregates was clearly distinct from that of Tau tangles. Approximately 20% of neurons were Bax∆2-positive and about 5% were Tau-positive. Only about 3% of total neurons were double-positive for both Bax∆2 aggregates and Tau tangles, and no apparent colocalization was detected (Figure 4b,c). Consistently, by further analyzing levels of hyperphosphorylated Tau (T181 and T205, the most common markers in AD pathogenesis), we found that there was no detectable colocalization between Bax∆2 and either T181 or T205 P-Tau proteins (Figure 4d). We further examined whether the presence of Bax∆2 protein increased Tau aggregation, and there was no detectable change in Tau aggregation levels seen in examined human tissues (Figure S7) and in Bax∆2 transfected mouse neuronal HT22 cells (Figure S8). These results suggest there is no physical relationship between Bax∆2 aggregates and Tau tangles, and they appear to be independent of each other in AD neurons.

### 3.4. Bax∆2 Aggregates Coexist and Colocalize with Stress Granules in AD Neurons

**Figure 5:**
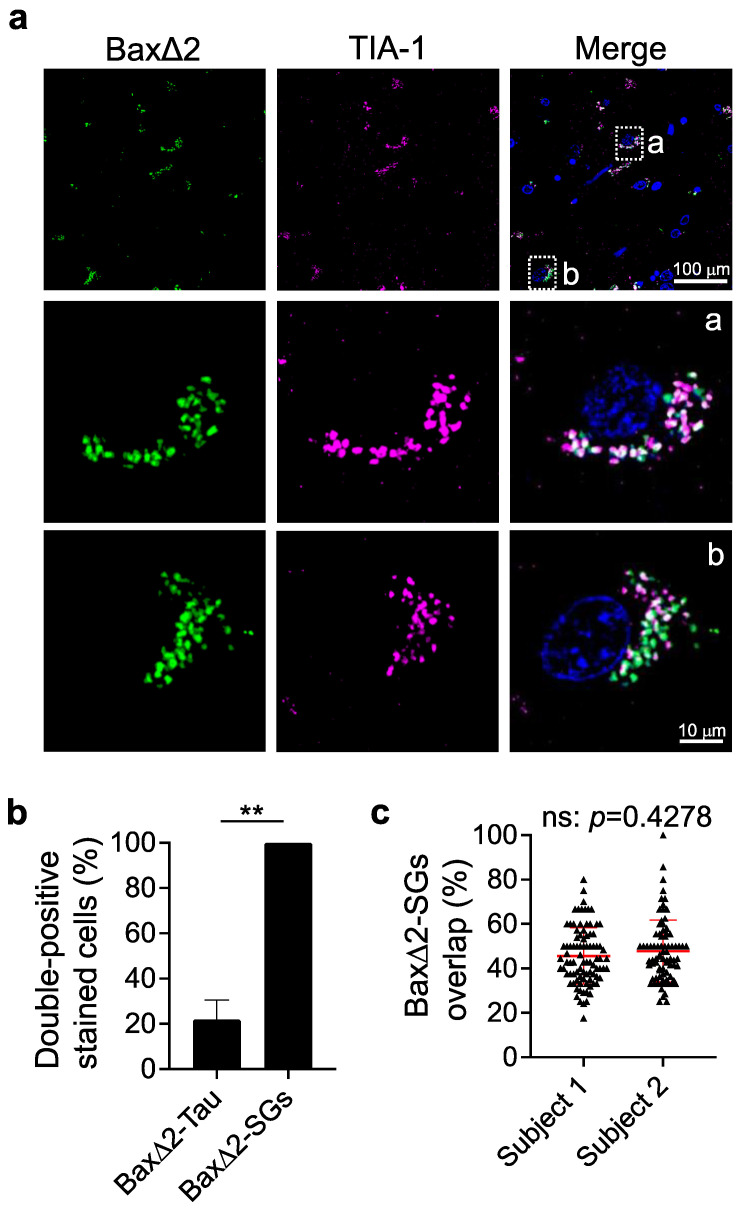
Intracellular localization of Bax∆2 aggregates in relationship to stress granules (SGs) in Alzheimer’s brain tissue. (**a**) Co-immunofluorescence staining of AD frontal lobe tissues with anti-Bax∆2 (green) and anti-TIA-1 stress granular marker (purple) antibodies (85-year-old female was shown). Nuclei were stained with DAPI. The enlarged regions in the boxed areas (a and b) are shown below (second and third row). Scale bars, 100 μm (first row), 10 μm (second and third rows). (**b**) Quantitation of double-positive neurons for Bax∆2-SGs and Bax∆2-Tau in human frontal lobe tissue. (**c**) Quantitation of positive-stained particle overlapping for Bax∆2 and SGs in individual cells from two AD patients (subject 1: 89 cells counted from the 75-year-old male AD patient; and subject 2: 80 cells counted from the 85-year-old female AD patient). The quantitation results were analyzed using the Mann–Whitney test. ns, not statistically significant. **, *p* < 0.01.

Stress granules (SGs) are dynamic large intracellular RNA-protein complexes involved in RNA processing, misfolded protein sequestration, and AD pathogenesis [42,43]. It is possible that stress granules are involved in the generation of Bax∆2 and/or sequestration of Bax∆2 protein aggregates. To determine whether the accumulation of Bax∆2 aggregates is associated with SGs, we co-immunostained the Bax∆2 of AD individuals with the SG marker TIA-1. Indeed, SG-positive staining was strongly detected in Bax∆2-positive cells (Figure 5a) with all Bax∆2-positive cells being SG-positive; furthermore, all SGs-positive cells were Bax∆2-positive, in striking contrast to the independent-like relationship between Bax∆2 and Tau (Figure 5b, the SGs staining controls shown in Figure S9). By further evaluating the individual SGs particles in relationship with Bax∆2 aggregates within each cell, we found that the extent of Bax∆2-SG double-positive particles varied widely (20–100%) (Figure 5c). These data suggest that the physical and functional relationship between Bax∆2 aggregates and SGs might be dynamic or depend on the cellular stress status in AD neurons.

### 3.5. Aggregation of Bax∆2 Is Critical for SG Formation, and the Bax∆2 C-Terminal Tail Is Required for Bax∆2-SG Colocalization and Cell Death

**Figure 6:**
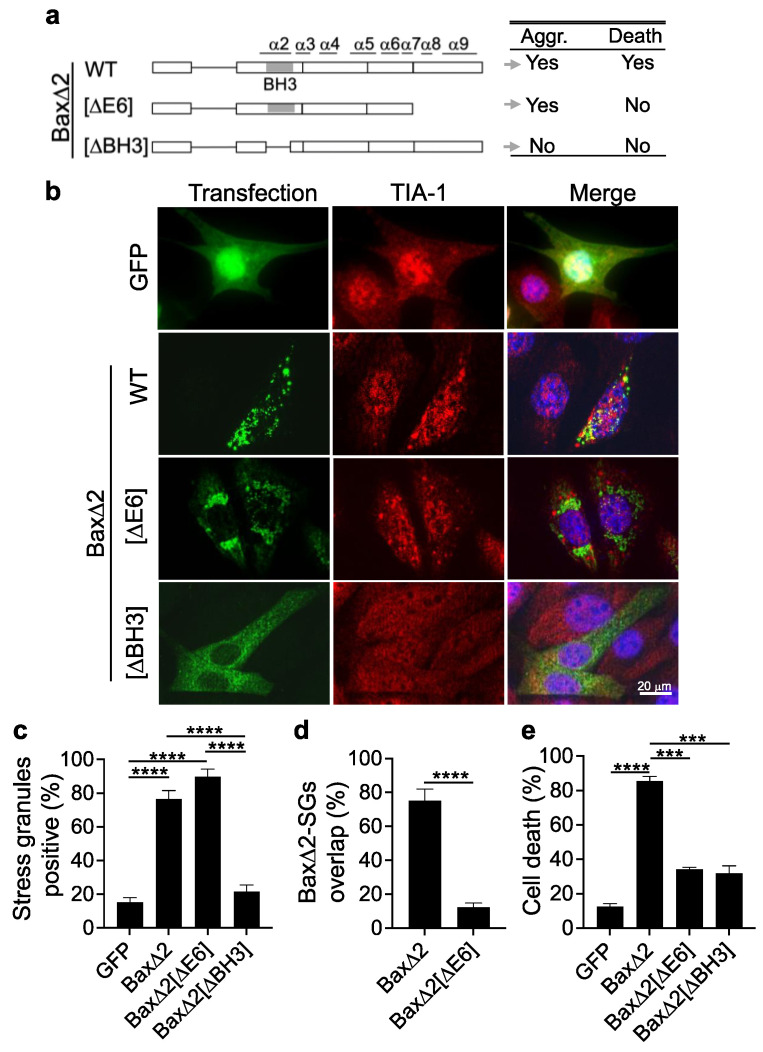
Bax∆2 functional domains for protein aggregation, SGs relationship, and cell death. (**a**) Schematic summary of Bax∆2 functional domains. Bax∆2 [∆E6], deletion of exon 6; Bax∆2 [∆BH3], deletion of the Bcl-2 homology domain 3. E1–6 indicated on the bottom represents the exons. α1–9 indicated on the top represent the helices. The behaviors of these mutants are listed on the right panel. Aggr., aggregation. (**b**) HT22 moue hippocampus neuronal cells were transfected with GFP, Bax∆2 and its mutants (**a**) followed by the immunofluorescent staining of Bax∆2 (green) and the SGs marker TIA-1 (red). (**c**) Quantitation of stress granule formation in (**b**) experiments. (**d**) Quantitation of positive-stained particle overlap between Bax∆2 or Bax∆2[∆E6] and for SGs in (**b**) experiments. (**e**) Cell death assay of HT22 cells transfected for 48h with the constructs indicated. All values are presented as the mean ± standard deviation (SD). The Mann–Whitney test was used to compare two groups (**d**). Comparisons of more than two groups were analyzed using one-way ANOVA followed by Tukey’s multiple comparisons test for (**c**,**e**). ***, *p* < 0.001, ****, *p* < 0.0001.

We previously demonstrated in cancer cells that the core region of Bax∆2 encoded by exons 3–5 was critical for protein aggregation and the disruption of the functional domain in the core region led to a diminished Bax∆2 capacity to aggregate. The C-terminal tail (mostly helix α9) was not required for protein aggregation but critical for the recruitment of caspase 8 and cell death [19] (Figure 6a). We wondered whether these Bax∆2 functional domains were also essential for the Bax∆2 behavior found in neurons. Therefore, we transfected various Bax∆2 mutant constructs in HT22 neuronal cells that lack endogenous Bax∆2. The results showed that the deletion of the C-terminal tail (∆E6) did not affect Bax∆2 aggregation or SGs formation (Figure 6b,c) but did disrupt the colocalization between Bax∆2 and SGs (Figure 6b,d). Deletion of the BH3 domain in the core region abolished both Bax∆2 aggregate and SG formation (Figure 6b,c). Consistently, Bax∆2-induced cell death was diminished in the absence of either the C-terminal tail or the core region BH3 domain (Figure 6e). These results indicate that SG formation was triggered by Bax∆2 aggregates, and the BH3 domain of Bax∆2 was essential for the formation of aggregates. However, the Bax∆2 aggregation without the helix α9 was not sufficient to trigger cell death. This indicates that the Bax∆2 C-terminal tail is not required for aggregation but is essential for the interaction with SGs and to execute neuronal cell death.

### 3.6. Bax∆2 Aggregates Prefer SGs and Not Caspase 8 to Induce Cell Death in Neurons

**Figure 7:**
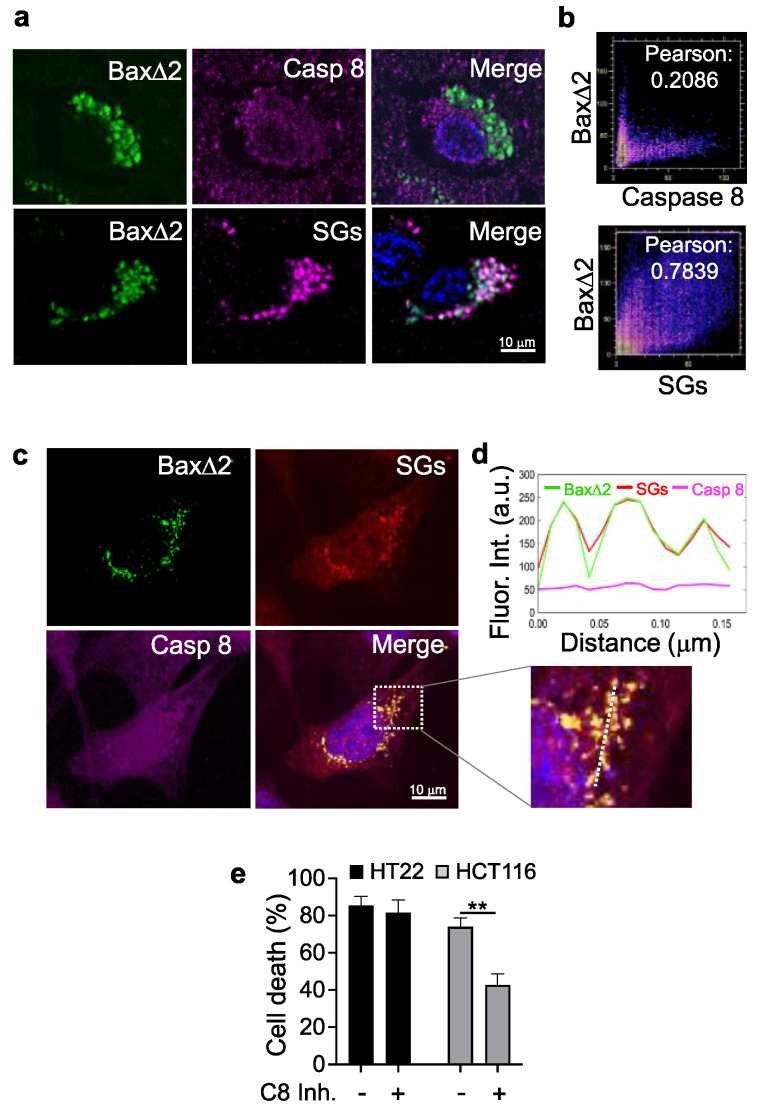
Target selectivity of Bax∆2 aggregates for SGs and caspase 8 in neuronal cells. (**a**) Co-immunofluorescence staining of Bax∆2 with either caspase 8 or SGs (TIA−1 marker) as indicated. Nuclei were stained with DAPI. (**b**) Quantitation of colocalization of Bax∆2 with caspase 8 and Bax∆2 with SGs from (A) using Image J. Pearson numbers indicate the correlation coefficient between the X (caspase 8 or SGs) and Y (Bax∆2) axes. (**c**) HT22 mouse hippocampus cells were transfected with Bax∆2 followed by immunofluorescent staining for Bax∆2 (green), SGs (red), and caspase 8 (purple) as indicated. The boxed area is enlarged in (**d**) for a line scan analysis of fluorescence intensity. (**e**) Cell death assay of HT22 cells and Bax-negative colon cancer HCT116 cells 48h after transfection with Bax∆2 in the presence and absence of a caspase 8 inhibitor. **, *p* < 0.01.

The Baxα C-terminal tail contains a transmembrane domain for anchoring on mitochondria. When Bax proteins fail to target mitochondria and form aggregates, these tails are accessible for binding to the caspase 8 N-terminal death effector domain in cancer cells [21]. We demonstrated that the α9 helical structure rather than the primary sequence was critical for Bax∆2 binding to caspase 8 [19]. In AD neurons, Bax∆2 aggregates colocalized with SGs, and the Bax∆2 C-terminus seemed to be required for such interaction as well (Figure 6). Could Bax∆2 interact with both SGs and caspase 8 in neurons? To determine this, we first examined the cellular localization of Bax∆2, caspase 8, and SGs in AD tissue by co-immunostaining. The results showed that the caspase 8 protein appeared to be spread throughout the cytosol, and there was no visible colocalization of Bax∆2 with caspase 8, in contrast to the strong colocalization of Bax∆2 with SGs in AD neurons (Figure 7a,b). Consistently, triple-coimmunostaining of Bax∆2, caspase 8, and SGs in transfected HT22 mouse neuronal cells confirmed this observation (Figure 7c,d). These results imply that Bax∆2 aggregates may rely on SGs rather than caspase 8 for cell death. Indeed, the cell death assay results supported this notion, as a caspase 8 inhibitor did not block Bax∆2-mediated cell death in these neuronal cells, in contrast to cancer cells (Figure 7e), suggesting that Bax∆2-SG-triggered cell death may not occur via caspase 8 in neuronal cells.

**Figure 8:**
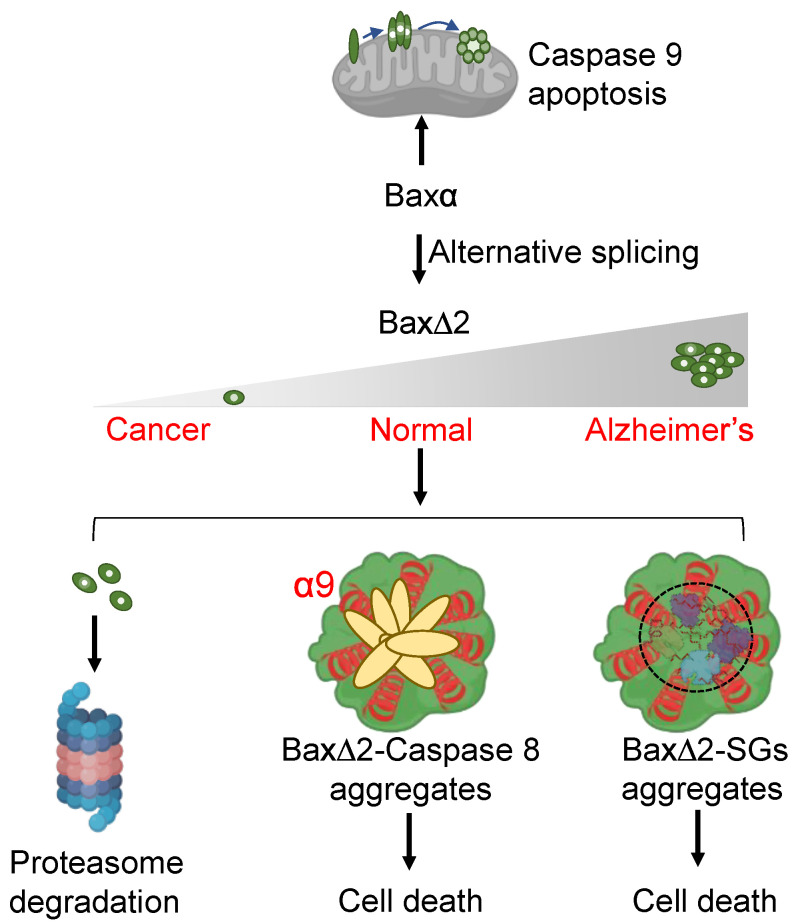
Graphic summary of Bax∆2 pro-death behaviors in different diseases. Alternative splicing of Baxα generates Bax∆2, which cannot target mitochondria but aggregates in the cytosol to trigger cell death. Cancer cells can avoid this cell death by active proteasomal degradation. However, in Alzheimer’s neurons, increased Bax∆2 protein amplifies the accumulation of aggregates. Unlike in cancer cells, in which Bax∆2 aggregates use its C-terminal tail (α9 helix) to recruit caspase 8 for cell death, Bax∆2 aggregates appear to use the same functional domain but instead target SGs for cell death in neurons.

In summary, unlike Baxα, Bax∆2 is unable to target mitochondria and instead forms protein aggregates in the cytosol. Baxα and Bax∆2 rely on the same domains for aggregation and cell death. However, the “plasticity” of Bax-mediated cell death is determined by its C-terminal tail by either anchoring onto mitochondrial outer membrane for classical apoptosis, recruiting caspase 8 for mitochondria-independent cancer cell death, or interacting with stress granules for caspase 8-independent cell death (Figure 8).

## 4. Discussion

Here, we report for the first time the behavior of Bax∆2 in neurons and its association with AD pathogenesis. Bax exon 2 alternative splicing frequently occurs in certain cancers [12], but had never been explored in neurogenerative diseases. Transcript analysis has shown that the brain expresses more alternatively spliced transcripts than other tissues and is influenced by aging-related neurodegenerative diseases such as AD [44]. Here, we found a high prevalence of Bax exon 2-spliced transcripts in AD patients (85%) (Figure 1b). Whether such a high frequency of Bax alternative splicing is AD-specific or common to other neurodegenerative diseases remains to be explored. Interestingly, the splicing level in the NCI group was also higher (48%) than expected. As the NCI groups were also elderly individuals (>70 years), it is possible that such Bax alternative splicing is driven by aging and amplified by certain pathological conditions. However, we previously detected strong Bax∆2-positive staining in the cerebellar Purkinje cells from several young and healthy individuals [32], suggesting possible non-pathological functions of Bax∆2 in the brain. Furthermore, whether the behavior of Bax∆2 aggregates observed in AD is similar in other neurodegenerative diseases also remains to be explored.

Endogenous Bax∆2 protein is very unstable and susceptible to proteasomal degradation in cancer cells, and treatment with the proteasomal inhibitors increased Bax∆2 protein levels and promoted cell death [18]. Although increased Bax alternative splicing in AD patients (Figure 1b,c) may partially contribute to the Bax∆2 protein production, it is unlikely as a predominant source of the aggregates observed here. It has been shown that the accumulation of aberrant forms of certain proteins such as APP^+1^ inhibited the proteasomal activity, which further promoted the accumulation and aggregation of additional proteins [45]. Moreover, association with SGs possibly protect Bax∆2 protein from proteasomal degradation. All of these factors could enhance the stability of Bax∆2 protein and facilitate aggregate formation in neurons.

The interaction of Bax∆2 with caspase 8 occurs at the interface between the Bax∆2 helix α9 and caspase 8 N-terminal death effector domain (DED) [19,21]. Here, we detected the co-localization of Bax∆2 and SGs (Figure 5). It is important to mention that mainly the secondary structure of the helix α9 helix, and not the primary sequence, is critical for Bax∆2-caspase 8 binding [19]. This conclusion is based on the successful interaction between Bax∆2ω (another Bax isoform) and caspase 8. The amino acid sequence of Bax∆2ω is the same as Bax∆2 up to exon 5 but has an additional retention of intron 5 that results in a different C-terminal amino acid sequence [11]. However, the C-terminal helical structures of both isoforms are surprisingly consistent and well aligned [19]. Therefore, the secondary structure rather than primary sequence requirement might provide great flexibility for Bax∆2 to choose its targets. Unlike a single caspase 8 protein, SGs are composed of many components, including RNAs and many different proteins [46]. It is likely that Bax∆2 C-terminal helices interact with more than one substrate in the SGs complex.

Surprisingly, although the Bax∆2 functional domains for aggregation and cell death in cancer and neuronal cells are the same, the death execution target appears to be selective (Figure 7). Why does Bax∆2 rely on SGs instead of caspase 8 to trigger cell death in neurons? This might be due to the prolonged stress in AD development. Initial recruitment of Bax∆2 aggregates into SGs may be for cell survival. Once the Bax∆2 C-terminal tail is occupied by the SGs component, it might not be available for binding and activating caspase 8. As the disease progresses, chronically persistent “pathological SGs” can mature into more stable complexes as a breeding ground of AD “seeds” and ultimately lead to cell death [42,43]. Which components in SGs interact with Bax∆2 and how it causes cell death remains to be explored.

SGs are dynamic cytoplasmic aggregates of RNA and proteins that form in response to various stress stimuli, including oxidative stress, heat shock, and viral infections [46]. SGs contain RNA and a growing list of various proteins, such as the RNA-binding protein TIA-1 (T-cell intracellular antigen 1), TDP-43 (TAR DNA-binding protein 43), and DDX3X (DEAD-Box helicases 3 X-linked). Their assembly and disassembly are highly dynamic when coupled with translational control and RNA metabolism [46], and they have been implicated in the pathogenesis of many diseases, particularly neurodegenerative diseases [42,43]. SGs can be regulated by autophagy [46,47,48,49], a process by which cells degrade and recycle damaged organelles and proteins [50]. There is a lack of evidence that SGs can directly trigger apoptotic cell death. However, SGs can indirectly contribute to apoptosis by interfering with cellular processes, such as translation disruption and oxidative stress [46]. Additionally, some studies suggested that chronic stress granules can recruit apoptotic regulatory proteins, such as MAPK (mitogen-activated protein kinases), promoting apoptosis [51]. It is possible that chronic SG-mediated cell death here (Figure 6e) is dependent on the cellular context and pathological conditions for different types of disease.

## 5. Conclusions

The discovery of Bax∆2 behavior in AD neurons broadens our vision beyond conventional sources of protein aggregates. Many membrane proteins have oligomerization properties, and off-target effects resulting from various pathological conditions at the genetic, transcriptional, translational, or protein levels could potentially create a new source of protein “seeds”. The accumulation of these seeds can trigger the formation of stress granules and neuronal stress. Chronic stress over the long term can convert stress granules from a pro-survival to pro-death mode, ultimately contributing to AD pathogenesis.

## Data Availability

The results published here are in part based on data obtained from the AD Knowledge Portal (https://adknowledgeportal.org) (accessed on 20 March 2023). ROSMAP resources can be requested at https://www.radc.rush.edu (accessed on 20 March 2023). The custom script get_BAX.pl is available at the following link: https://github.com/annecarolm/Publication_scripts/tree/main/2023_eLife_Yao_Bax_Alzheimers (accessed on 20 March 2023).

## Supplementary Materials

The following supporting information can be downloaded at: https://www.mdpi.com/article/10.3390/biom13060970/s1, Figure S1: Information of human brain RNA−seq databases; Figure S2: Bax∆2 antibody validation; Figure S3: Information of human brain tissues; Figure S4: Correlation between Bax∆2 positivity and AD patients’ age; Figure S5: Immunostaining of Bax∆2 protein in different brain regions from a 73−year−old male AD patient; Figure S6: Immunostaining of Bax∆2 proteins in different regions of human normal brains; Figure S7: Correlation between Bax∆2 and Tau positivities; Figure S8: Correlation between Bax∆2 aggregate and Tau tangle in transfected cells; Figure S9: Immunofluorescent staining SGs of non-AD and AD human brain tissue.
