# Supplementary material for: Unconventional Source of Neurotoxic Protein Aggregation from Organelle Off-Target Bax∆2 in Alzheimer’s Disease
Source: Biomolecules. 2023 Jun 10;13(6):970. doi: 10.3390/biom13060970 (PMC10296721; doi:10.3390/biom13060970)
Supplement: Supplementary file 1 [file biomolecules-13-00970-s001.zip › biomolecules-2416445-supplementary.pdf]

Supplementary Information

Unconventional Source of Neurotoxic Protein Aggregation from Organelle Off-Target BaxΔ2 in Alzheimer's Disease

Biomolecules

Qi Yao 1, Anne Caroline Mascarenhas dos Santos 1, Huaiyuan Zhang 1, Adriana Mañas 2, Ammarah Hussaini 1, Ujin Kim 1, Congtai Xu 1, Sana Basheer 1, Shinya Tasaki 3 and Jialing Xiang 1,*

[1]Department of Biology, Illinois Institute of Technology, Chicago, IL 60616, USA
[2] Department of Laboratory Medicine, Lund University, 22381 Lund, Sweden
[3] Rush Alzheimer's Disease Center, Rush University Medical Center, Chicago, IL 60612, USA

*Corresponding author:

Jialing Xiang

Department of Biology

Illinois Institute of Technology

3101 South Dearborn Street, Chicago, IL 60616, USA

Phone: (312)-567-3491

Fax: (312)-567-3494

Email: xiang@iit.edu

Fig. S1

Human brain RNA-seq databases

Data ID	Data type	Female (%)	AD (N)	NCI (N)
syn8612097	RNA-seq	64%	253	201
syn24175555	RNA-seq	75%	71	126

Note: AD, Alzheimer's disease; NCI: None Cognitive impair

Fig. S2

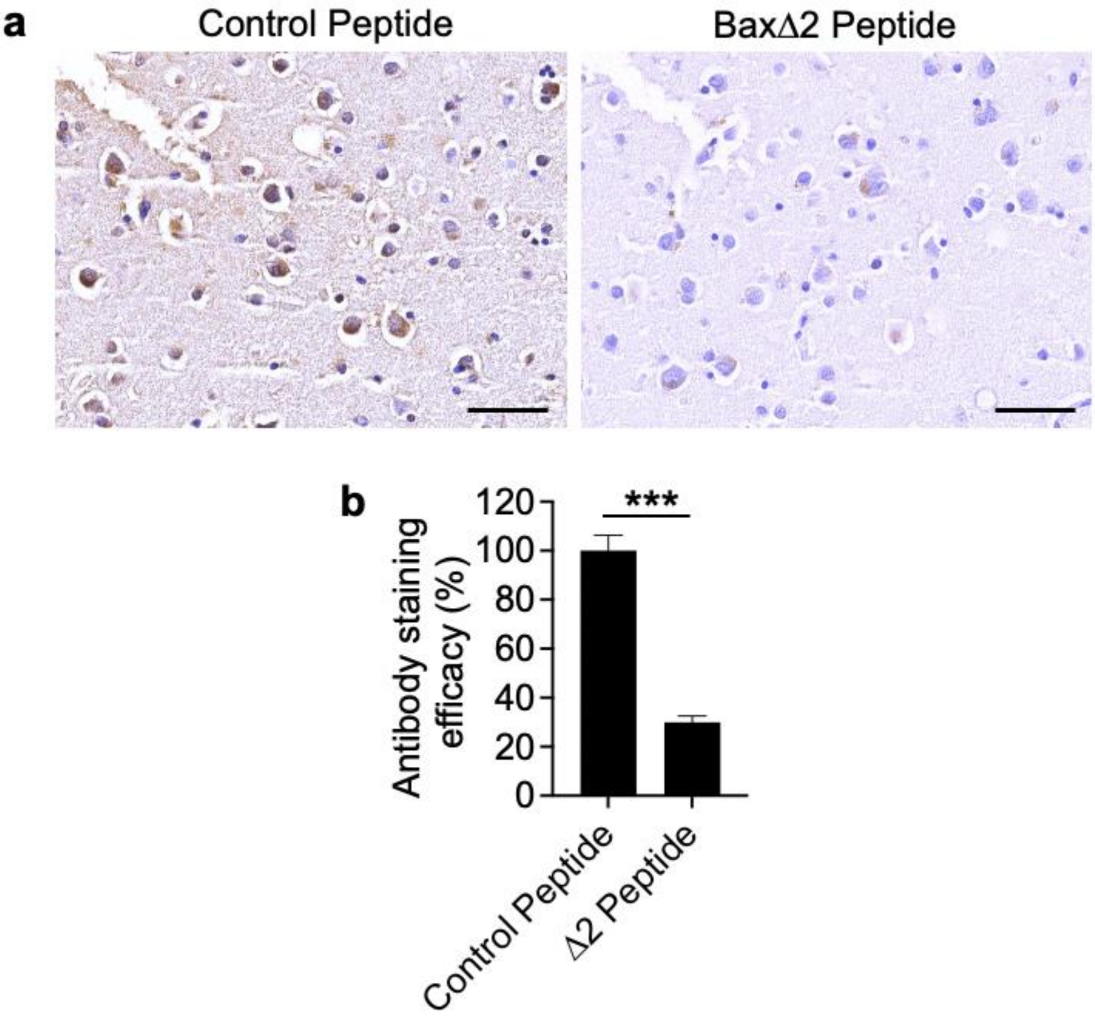

Caption: a Immunostaining of human brain tissues with anti-BaxΔ2 antibody in the presence of Baxα (control) or BaxΔ2 antigen peptide. Scale bars, 50 μm. **b** Quantitation of (a) using Cell-Profiler software and analyzed by non−parametric statistical analysis, *** $p < 0.001$

Fig. S3

Human Brain Tissue information

Patient	Source	Gender	APOE genotype	Age death	Braaksc	Cogdx
1	RADC	M	33	77	2	1
2	RADC	F	33	87	4	1
3	RADC	F	33	79	3	1
4	RADC	F	33	82	1	1
5	RADC	M	33	72	0	1
6	Biochain	M	N/A	54	N/A	1
7	Biomax	M	N/A	38	N/A	1
8	Biomax	F	N/A	18	N/A	1
9	Biomax	M	N/A	15	N/A	1
10	RADC	M	33	88	4	4
11	RADC	F	33	90	3	4
12	RADC	F	34	86	5	4
13	RADC	F	33	89	4	4
14	RADC	F	NA	78	5	4
15	RADC	M	34	87	5	4
16	RADC	M	34	89	6	4
17	RADC	F	44	83	5	4
18	RADC	M	33	86	5	4
19	RADC	M	23	89	4	4
20	Biochain	M	N/A	73	N/A	4
21	Biochain	F	N/A	85	N/A	4
22	Biochain	M	N/A	88	N/A	4

Note: RADC, Rush Alzheimer's Disease Center.
Braaksc, Braak stages: stage 1 and 2 indicate NFTs confined mainly to the entorhinal region of the brain; stage 3 and 4 indicate involvement of limbic regions such as the hippocampus; stage 5 and 6 indicate moderate to severe neocortical involvement.
Codgx, Clinical Consensus Diagnosis, 1 = non-AD, 4 = AD.

Fig. S4

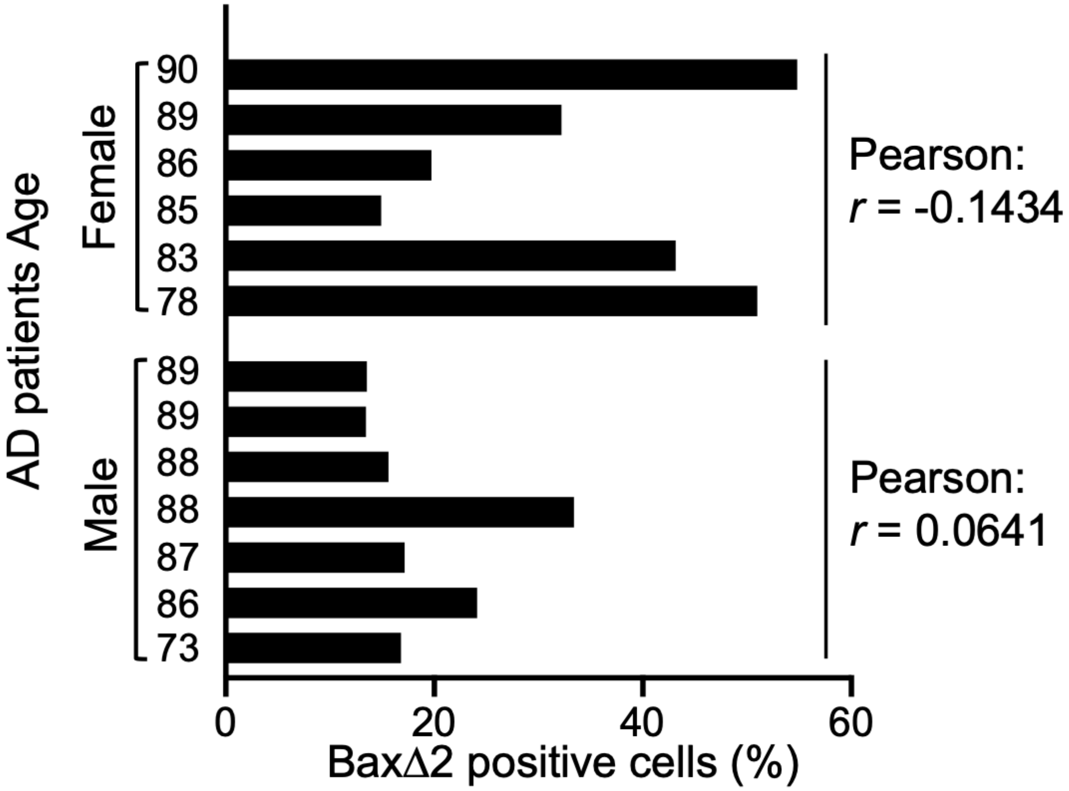

Caption: Correlation histogram graph of BaxΔ2 positivity and AD patients' age. Pearson numbers indicate the correlation between the X (BaxΔ2 positive cells) and Y (AD female or male patients' age) axes.

Fig. S5

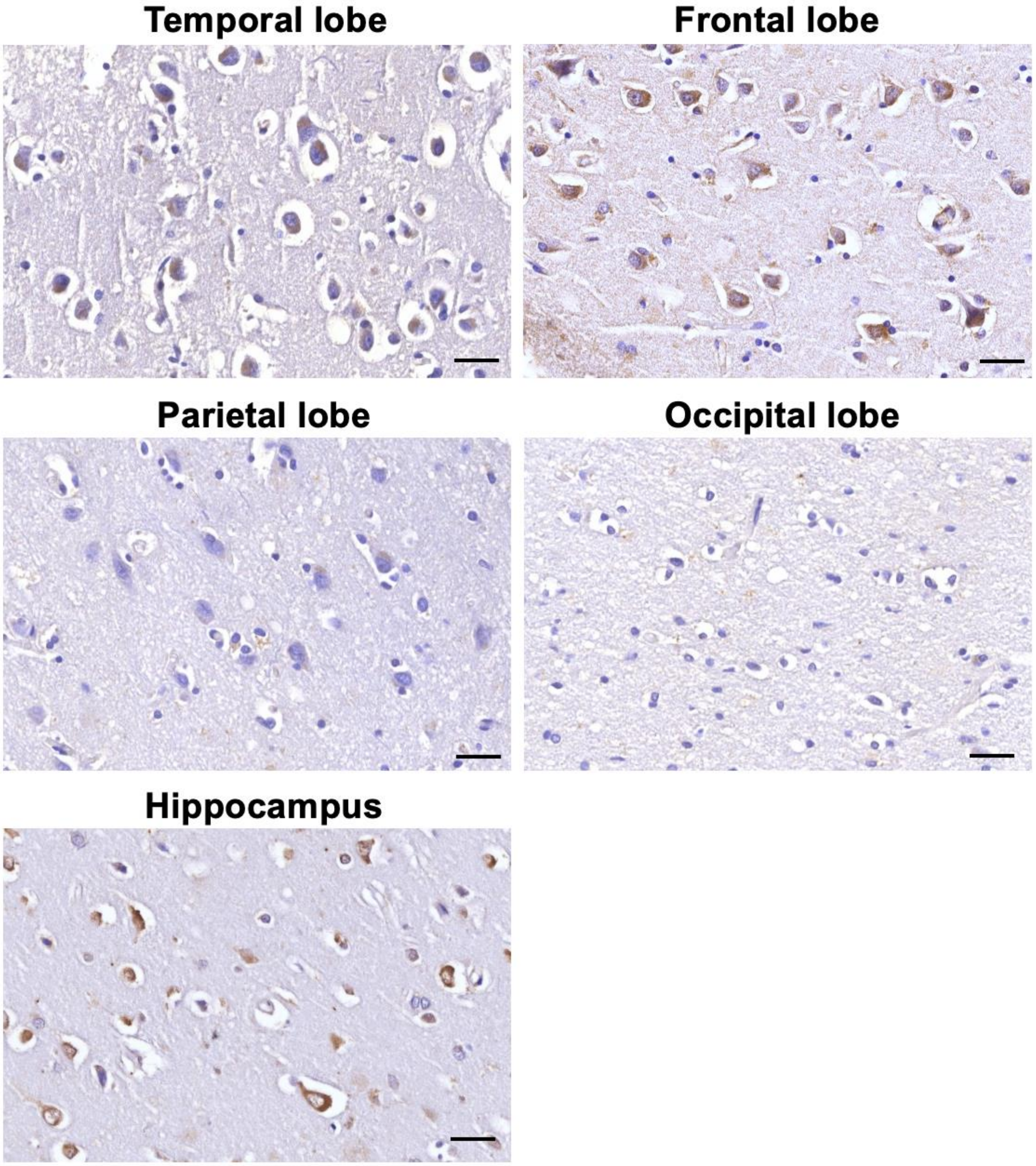

Caption: Immunostaining of BaxΔ2 proteins in different brain regions from a 73−year−old male AD patient. Scale bars, 50 μm.

Fig. S6

a

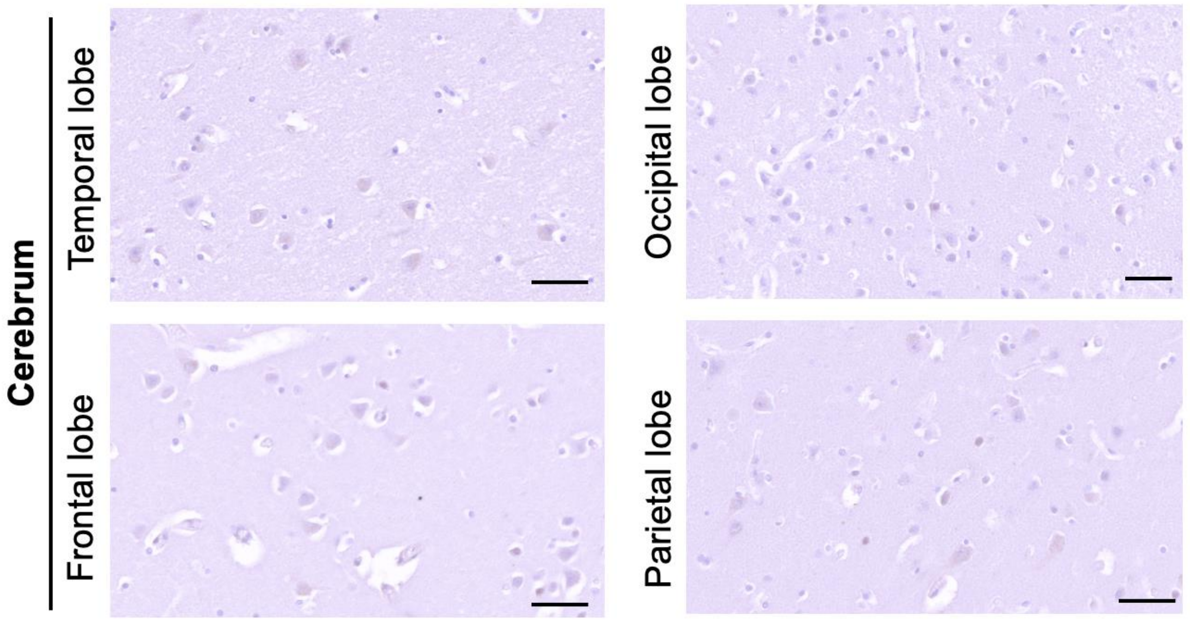

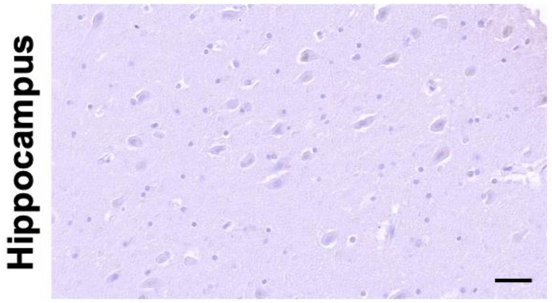

b

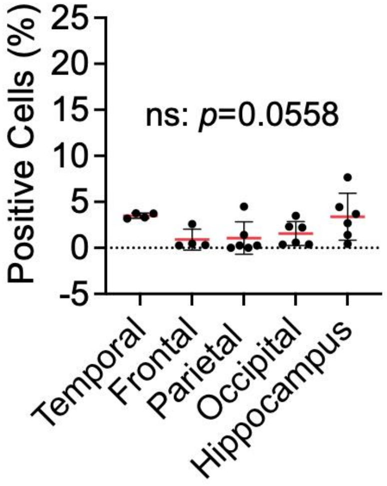

c

Anatomic site	Age	Sex
Temporal lobe	45	F
Temporal lobe	27	M
Frontal lobe	18	F
Frontal lobe	38	M
Parietal lobe	45	M
Parietal lobe	23	F
Parietal lobe	54	M
Occipital lobe	23	M
Occipital lobe	45	F
Occipital lobe	27	F
Hippocampus	47	M
Hippocampus	28	F
Hippocampus	41	M

Caption: a Immunostaining of BaxΔ2 proteins in different regions of human normal brains. scale bars, 50 µm. **b** Quantitation of (a). **c** Tissue source information for (a) experiments.

Fig. S7

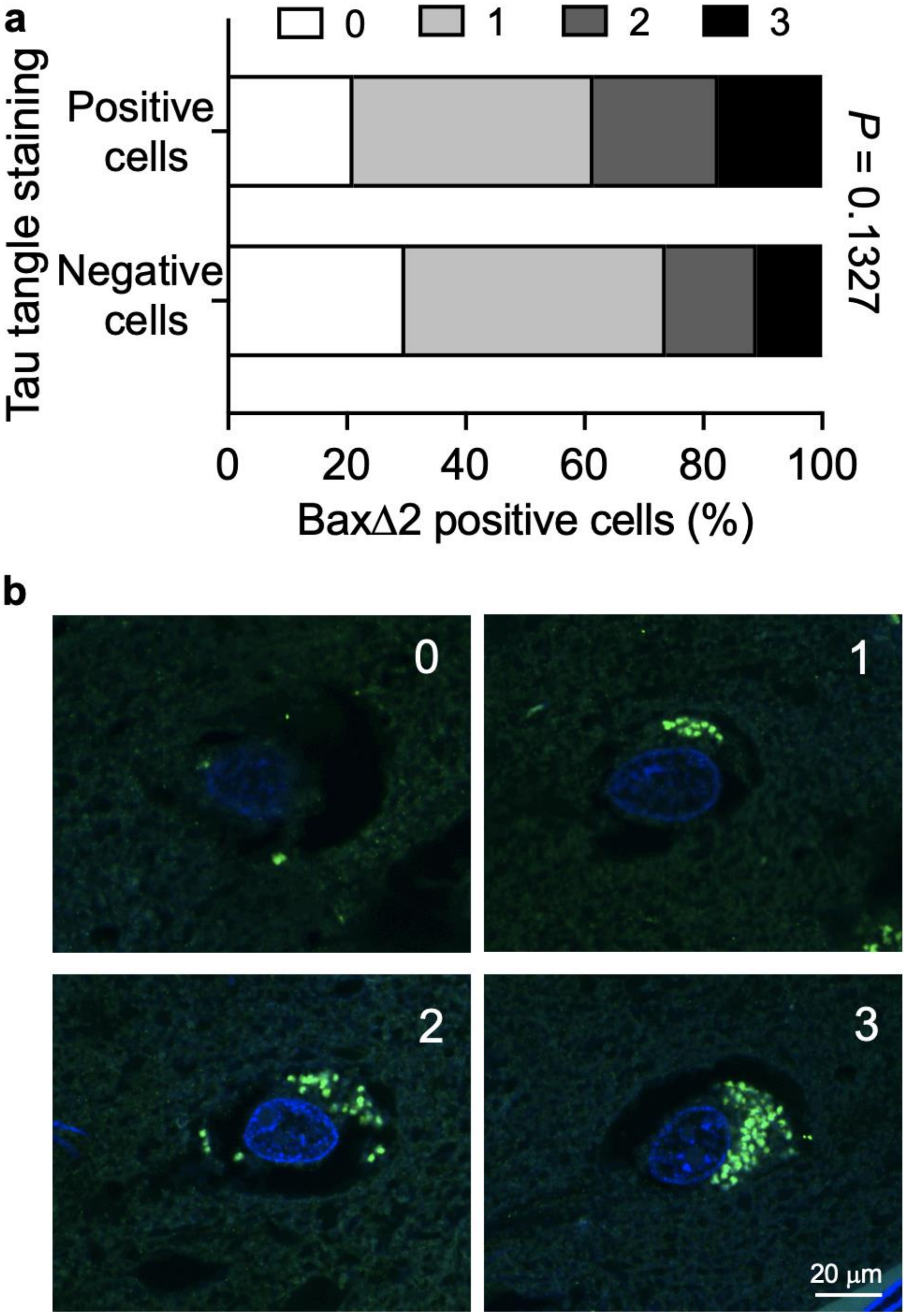

Caption: a Quantitation of correlations between BaxΔ2 positive cells and Tau positivity from stained tissue samples in Figure 4. Tau−positive cells (n=60) and Tau negative cells (n=60) were analyzed in the quantitation. BaxΔ2 positivity was classified into 4 different levels: 0, 1, 2, 3. Typical images for each score are shown in (**b**). Parametric statistical analysis was performed, *p* = 0.1327.

Fig. S8

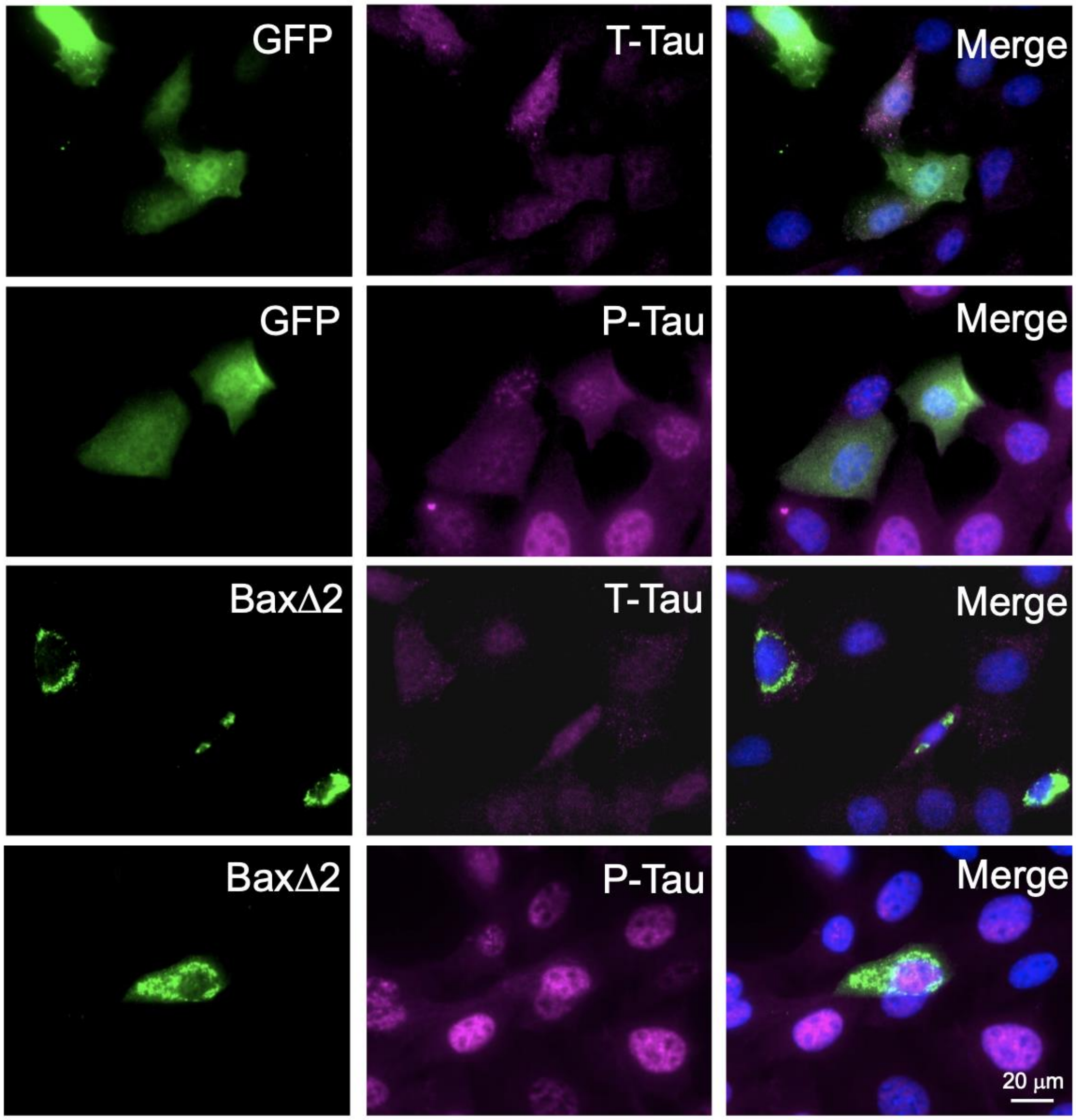

Caption: HT22 cells were transfected with GFP, BaxΔ2 followed by immunofluorescent staining for BaxΔ2 (green) and T−Tau or P−Tau (T205) (purple), Nucleus was stained as blue. Scale bars, 20 μm.

Fig. S9

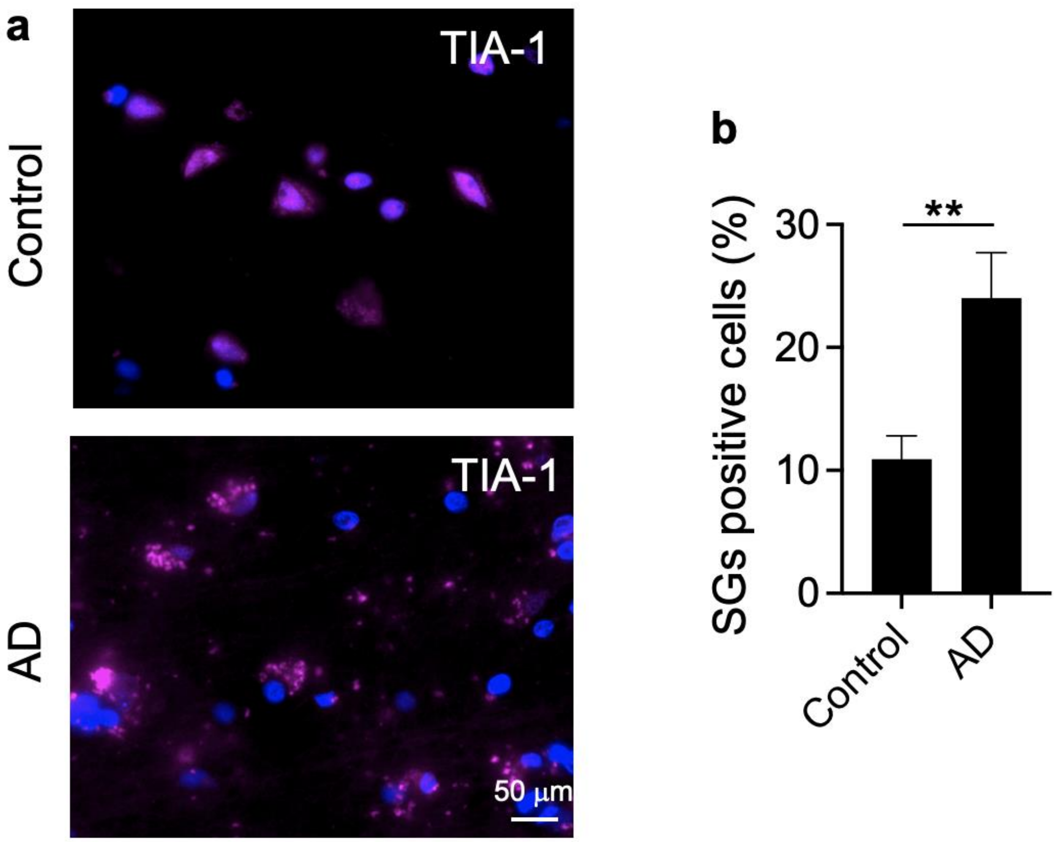

Caption: a Immunofluorescent staining of non−AD and AD human brain tissues with SGs marker TIA−1 (purple). Scale bars, 50 μm. **b** Quantitation of (a) analyzed by parametric statistical analysis, ** $p < 0.01$
